# Severe paradoxical reaction in tuberculous meningitis

**DOI:** 10.1016/j.idcr.2020.e01009

**Published:** 2020-11-17

**Authors:** Joseph Donovan, Nguyen Truc Thanh, Guy E. Thwaites, Nguyen Hoan Phu

**Affiliations:** a Oxford University Clinical Research Unit, Centre for Tropical Medicine, Ho Chi Minh City, Viet Nam; b Centre for Tropical Medicine and Global Health, Nuffield Department of Medicine, University of Oxford, United Kingdom; c Hospital for Tropical Diseases, Ho Chi Minh City, Viet Nam; d Vietnam National University School of Medicine, Ho Chi Minh City, Viet Nam

**Keywords:** Tuberculous meningitis, Paradoxical reaction

## Abstract

A 31-year-old female presented with a 3-week history of fever and headache. CSF Ziehl-Neelsen smear microscopy revealed acid-fast bacilli, and CSF GeneXpert MTB/RIF was positive for *Mycobacterium tuberculosis* with no mutations of rifampicin resistance. Tuberculous meningitis (TBM) was diagnosed. Baseline contrast-enhanced brain magnetic resonance imaging (MRI) was unremarkable. Eight weeks later the patient developed markedly reduced visual acuity and clinical signs consistent with left 3rd and 6th cranial nerve palsies. Repeat contrast-enhanced brain MRI revealed extensive tuberculous exudate filling the basal cisterns of the brain consistent with a severe paradoxical reaction of TBM. High dose intravenous dexamethasone was administered, with visual acuity returning to near-normal over 3–4 weeks. In TBM paradoxical inflammatory reactions are common yet difficult to predict. When severe, they may result in substantial neurological morbidity and death. Prompt host directed therapies such as corticosteroids may reduce chances of permanent neurological damage.

## Case report

**Fig. 1:**
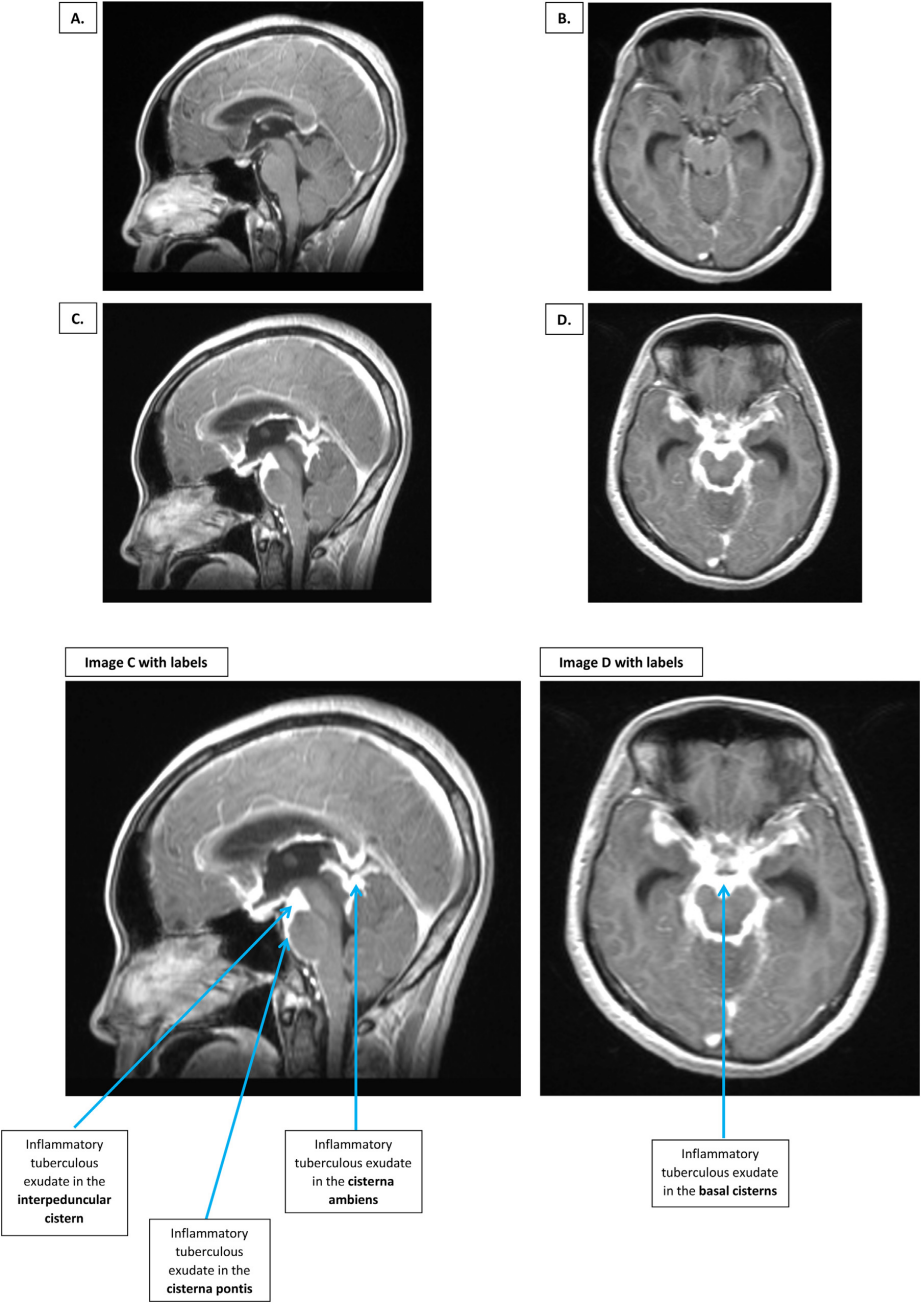
Sagittal and axial contrast-enhanced MRI images of a 31-year-old woman with TBM, before (**A & B**) and at the time of (**C & D**) a severe paradoxical reaction. Baseline sagittal (**A**) and axial (**B**) images show no neuroinflammatory features of TBM. Repeat imaging after 2 months shows extensive tuberculous exudate filling the cisterna pontis (anterior to the pons), interpeduncular cistern (anterior and superior to the pons), and cisterna ambiens (superior to the cerebellum), of the brain (C & D). Sagittal imaging (**C**) shows clear delineation between exudate-filled cistern and surrounding brain structures. Axial imaging (**D**) demonstrates a thick exudative ring with anterior projections, typical of basal exudate appearances in TBM. MRI = Magnetic resonance imaging. TBM = Tuberculous meningitis.

A 31-year-old female presented to the Hospital for Tropical Diseases (Ho Chi Minh City, Vietnam) with a 3-week history of fever and headache. There was no relevant past medical history. On examination she was confused (Glasgow coma score 13/15) with no focal neurological signs. Vital signs were unremarkable. A chest X-ray did not show appearances consistent with pulmonary or miliary tuberculosis. HIV testing was negative. Cerebrospinal fluid (CSF) parameters were as follows: white blood cells 676 cells/mm^3^ (83 % neutrophils), protein 1.64 g/L, glucose 0.47 mmol/L (paired serum glucose 5.8 mmol/L), and lactate 8.72 mmol/L. CSF Ziehl-Neelsen smear microscopy revealed acid-fast bacilli, and CSF GeneXpert MTB/RIF was positive for *Mycobacterium tuberculosis* with no mutations of rifampicin resistance. Tuberculous meningitis (TBM) was diagnosed, and first line anti-tuberculosis chemotherapy (rifampicin, isoniazid, pyrazinamide and ethambutol) was commenced. An 8-week tapering course of dexamethasone or placebo was administered (this was a double-blinded allocation in a host *leukotriene A4 hydrolase* genotype stratified clinical trial [1]). Baseline contrast-enhanced brain magnetic resonance imaging (MRI) was unremarkable (**images A & B**). Fully sensitive *M. tuberculosis* was cultured from CSF after 8 days. After four weeks of in-patient monitoring the patient was discharged home (Fig. 1).

Eight weeks later the patient returned to the same hospital, with markedly reduced visual acuity and clinical signs consistent with left 3rd and 6th cranial nerve palsies. Colour vision was unaffected. Repeat contrast-enhanced brain MRI revealed extensive tuberculous exudate filling the basal cisterns of the brain consistent with a severe paradoxical reaction of TBM (**images C & D**). High dose intravenous dexamethasone (initially 0.4 mg/kg/day, with tapering reduction over 8 weeks) was administered. The patient’s visual acuity gradually returned to near-normal over 3–4 weeks.

In TBM paradoxical inflammatory reactions are common yet difficult to predict. When severe, they may result in substantial neurological morbidity and death. Pathogenesis is uncertain however, they are often considered an excessive host immune response to dead or dying mycobacteria. [2,3] The wide spectrum of paradoxical reactions in TBM includes tuberculomas and arachnoiditis however, obstructive inflammatory basal exudate is highly specific [4]. Prompt host directed therapies such as corticosteroids may reduce chances of permanent neurological damage.

## Funding

JD and GET are supported by the Wellcome Trust, UK. The funder had no role in the writing or submission of this manuscript.

## Consent

Written informed consent was obtained from the patient for publication of this case report and accompanying images. A copy of the written consent is available for review by the Editor-in-Chief of this journal on request.

## Author contributions

JD drafted the manuscript. JD, NTT, GET and NHP all contributed to the care of the patient. All authors reviewed and approved the submission.

## Declaration of Competing Interest

The authors report no declarations of interest.
